# Inhibitory Effects of PC-SPESII Herbal Extract on Human Breast Cancer Metastasis

**DOI:** 10.1155/2013/894386

**Published:** 2013-06-25

**Authors:** Xiu-Feng Wang, Jia Du, Tian-Ling Zhang, Qian-Mei Zhou, Yi-Yu Lu, Hui Zhang, Shi-Bing Su

**Affiliations:** Research Center for Traditional Chinese Medicine Complexity System, Shanghai University of Traditional Chinese Medicine, 1200 Cailun Road, Pudong, Shanghai 201203, China

## Abstract

Cancer metastasis is refractory to most forms of chemotherapy. Conventional and alternative drugs, such as Chinese herbal remedies, have been developed to target metastatic cancer cells. In this study, we investigated the effects of PC-SPESII, an herbal formulation, on the migration, invasion, and metastasis of an experimental human breast cancer cell line *in vivo* and *in vitro*. PC-SPESII suppressed pulmonary metastasis and tumor growth of MDA-MB-231 human breast cancer xenografts without affecting body weight, liver function, and kidney function. PC-SPESII also inhibited MDA-MB-231 cell migration and invasion *in vitro* in a dose-dependent manner. Based on ELISA analysis, secretion of MMP-2 and MMP-9, proteins associated with extracellular matrix degradation, was reduced in response to PC-SPESII treatment. Western blot analysis of whole-cell extracts revealed that the levels of proteolytic proteins associated with matrix and base membrane degradation (MMP-2, MMP-9, and uPA) were decreased and the levels of their endogenous inhibitors (TIMP1 and TIMP2) were increased. Moreover, the p38MAPK and SAPK/JNK signaling pathway, which stimulates proteolytic enzymes and matrix degradation, was inhibited by PC-PSESII. Remarkably, cotreatment with PC-PSESII and p38MAPK or SAPK/JNK inhibitors magnified the antimetastatic phenotype. Our results indicate that PC-PSESII impairs human breast cancer metastasis by regulating proteolytic enzymes and matrix dynamics through the p38MAPK and SAPK/JNK pathway.

## 1. Introduction

Breast cancer is the most common cancer among women, with 1.38 million cases diagnosed in 2008. Incidence rates of breast cancer vary by geographic region. They were highest in Europe and lowest in Africa and Asia [1], although the rates in China are rapidly increasing [2].

Metastasis is the major cause of death in cancer patients. It is a multifaceted process that results from coordinated events including cancer cell invasion, migration, and adhesion [3]. Degradation of extracellular matrix (ECM) and basement membrane (BM) by proteolytic enzymes and subsequent cancer invasion are the essential early steps of metastasis [4]. Matrix metalloproteinases (MMPs) and urokinase-type plasminogen activator (uPA) are the two important proteolytic enzymes that degrade the ECM and BM. Accordingly, expression of MMP-2, MMP-9, uPA, and uPA receptor (uPAR) is associated with increased tumor-cell invasion and metastasis in breast cancer [5, 6].

The functions of mitogen-activated protein kinase (MAPK) pathways are abundant in cancer cell progression. These pathways have been implicated in cell proliferation, differentiation, apoptosis, angiogenesis, and tumor metastasis [7]. In recent years, studies have shown that MAPK signaling is important for malignant tumor development. In early stages of metastasis, MAPK signaling pathways help regulate tumor cell adhesion, motility and degradation of ECM and BM [7–11]. 

Today, chemotherapy is the most frequently used treatment for breast cancer and other cancers. However, this method of treatment is not selective for cancer cells and often leads to the destruction of normal cells [12]. To compensate for the limitations and toxicity of chemotherapy, Chinese herbal medicines and other alternative strategies are being developed. These agents are also being tested for their efficacy in preventing or suppressing metastasis. PC-SPESII, an herbal mixture, is made up of seven Chinese herbs (*Isatis indigotica, Glycyrrhiza glabra, Panax pseudoginseng, Rabdosia rubescens, Dendranthema morifolium, Scutellaria baicalensis, *and* Ganoderma lucidum*) [13]. This mixture contains 7 recognized and active antineoplastic compounds. A related mixture, PC-SPES, has an additional herb called saw palmetto. PC-SPES had been widely used for prostate cancer [14, 15] and PC-SPESII has been used in a phase I trial for prostate cancer [13]. The anticancer mechanisms of PC-SPES have been studied *in vitro *[16–21]. However, the inhibitory activities, if any, of PC-SPESII in the highly metastatic human breast cancer MDA-MB-231 cells have not been investigated. 

In this study, we investigated the effects of PC-SPESII on migration, invasion, and metastasis of MDA-MB-231 cells and its molecular mechanisms of action. We found that PC-SPESII inhibits MDA-MB-231 cell migration, invasion, and metastasis. Furthermore, PC-SPESII regulates MMPs and uPA proteolytic enzymes via the p38MAPK and SAPK/JNK signaling pathway. Remarkably, PC-SPESII has no side effects.

## 2. Materials and Methods

### 2.1. Reagents

Matrigel, 3-(4, 5)-dimethylthiahiazo(-z-y1)-3, 5-di-phenytetrazoliumromide (MTT), was from Sigma (St. Louis, MO, USA). The antibodies against MMP-9, MMP-2, TIMP-1, TIMP-2, uPA, and uPAR were obtained from Santa Cruz Biotechnology (Santa Cruz, CA, USA). p38MAPK, p-p38MAPK, p-ERK1/2, p-SAPK/JNK, and SAPK/JNK antibodies were obtained from Cell Signaling Technology (Boston, MA, USA). The p38MAPK inhibitor SB203580 and the SAPK/JNK inhibitor SP600125 were obtained from Biomol (Philadelphia, PA, USA). 

### 2.2. Drugs

**Figure 1:**
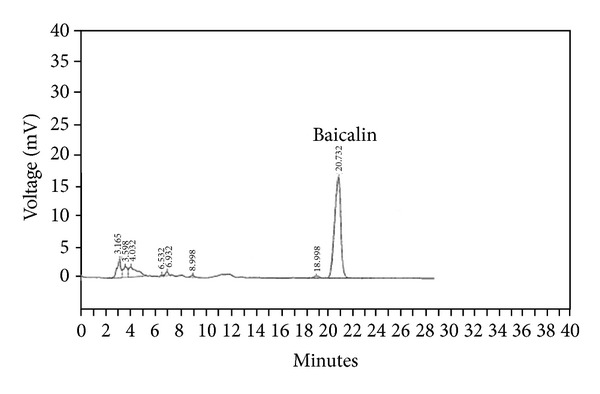
HPLC trace of the PC-SPESII extract. The major representative peaks of herbs in the formula are marked by their retention times. Baicalin represents one of markers listed in the PC-SPESII formula. The following HPLC conditions using a Discovery C18 analytical column (4.6 mm × 25 cm) were used: 100% methanol mobile phase at a flow rate of 1.0 mL/min and UV absorbance detected at 280 nm. Retention times of purified PC-SPESII are shown directly on this tracing.

PC-SPESII was obtained from Shanghai Zhong Yao BioTech Co., Ltd. (Shanghai, China). The following crude herbs, 1.0 g of Huangqin, 2.0 g of Daqingye, 1.0 g of Donglingcao, 0.5 g of Sanqi, 3.0 g of Lingzhi, 1.5 g of Juhua, and 0.5 g of Gancao, were made into PC-SPESII extract powder. The quality control and the standardization of each preparation of PC-SPESII is established and enforced strictly by Shanghai Zhong Yao BioTech Co., Ltd. To provide adequate quality control, the contents of major ingredients were measured on-line during the manufacturing processes (Table 1). The formulated PC-SPESII was subjected to high-performance liquid chromatography (HPLC) finger printing analysis in which the major peak was identified as the marker compound Baicalin (Figure 1).

320 mg of PC-SPESII powder was extracted with 70% ethanol as reported previously [16]. The ethanol extracts were kept at −20°C, and dilutions were made in the same culture media used for all *in vitro* studies. For *in vivo* studies, capsulated extracts were suspended in 1.5% CMC with 0.2% Tween 20 (Sigma, Chemical Co., St. Louis, MO, USA) as described previously [22].

### 2.3. Cell Culture

Human breast cancer MDA-MB-231 cells were obtained from American Type Culture Collection (Manassas, VA, USA) and were cultured in DMEM medium (Gibco, San Francisco, CA, USA) supplemented with 10% heat-inactivated (56°C, 30 min) fetal calf serum (PAA, A-4061, Pasching, Austria), 0.01 mg/mL insulin (Sigma, St. Louis, MO, USA), 2 mmol/L glutamine (Gibco, San Francisco, CA, USA), penicillin (100 U/mL), and streptomycin (100 *μ*g/mL). The cell culture was maintained at 37°C with 5% CO_2_ in a humidified atmosphere.

### 2.4. Human MDA-MB-231 Breast Cancer Xenograft Model Pulmonary Metastasis Assay

Female nude mice (6–8 weeks old) were purchased from the Laboratory Animal Center at Shanghai University of Traditional Chinese Medicine and housed in pathogen-free conditions throughout the duration of the experiment. Mice were given free access to commercial rodent feed and water. MDA-MB-231 cells (3 × 10^6^, suspended in 100 *μ*L of PBS) were injected into mammary fat pads of female athymic nude mice. One day after tumor cell inoculation, the mice were randomly divided into three groups (*n* = 8). In the treated group, 500 mg/kg of PC-SPESII was administered by oral gavage. Untreated groups were divided into a normal group and a model group (sham control) that were injected with physiological saline containing 1.5% CMC with 0.2% Tween 20. Body weight of each mouse was measured at different time points following tumor implantation. Mice were killed 2 months after tumor cell injection. The primary tumor of each mouse was weighed. The lungs were fixed with formalin. Thin sections were stained with hematoxylin and eosin. Five representative fields (at 100x magnification) for each group were photographed. The metastatic nodules of each field on the lungs were counted.

### 2.5. Kidney and Liver Function Tests

Blood was drawn from harvested eyeballs and centrifuged at 3000 rpm for 10 minutes to separate the serum. Glutamic oxalacetic transaminase (GOT/AST), glutamic pyruvic transaminase (GPT/ALT), serum creatinine (Cr), and blood urea nitrogen (BUN) were measured using the colorimeter testing kit (Kangcheng, Nanjing, China). Following the manufacturer's instructions, serum samples were measured at 510 nm, 510 nm, 510 nm, and 520 nm, respectively.

### 2.6. Cell Viability Assay

Cell viability was determined by MTT assay. MDA-MB-231 cells (5 × 10^4^ cells/mL) were seeded in 96-well culture plates. After overnight incubation, MDA-MB-231 cells were treated with various concentrations of PC-SPESII. Following incubation, cell growth was measured at different time points after the addition of 20 *μ*L MTT at 37°C for 4 h. Then, DMSO (150 *μ*L) was added to dissolve the formazan crystals. Optical density (OD) was measured at 490 nm with an ELISA plate reader (BioTek, Winooski, VT, USA).

### 2.7. Wound Healing Migration Assay

The wound healing migration assay was performed as reported previously [23]. MDA-MB-231 cells were seeded at a density of 1~5 × 10^5^ cells/well in 12-well culture plates and allowed to form a confluent monolayer. The layer of cells was then scraped with a 20–200 *μ*L micropipette tip to create a ~1 mm wide wound. Cells were then washed twice with fresh medium and replaced with FBS-free medium containing indicated concentration of PC-SPESII. After incubation at 37°C for 24 h and 48 h, cells were washed with PBS, fixed with 4% paraformaldehyde. Images of the wounds were captured at 0 h, 24 h, and 48 h after scraping at 100x magnification.

### 2.8. Migration and Invasion Assays

The *in vitro *cell migration and invasion assays were performed by using a Transwell chamber inserted with polyethylene terephthalate filter membrane containing 8 *μ*m pores in 24-well plates (Corning, USA) as reported previously [24]. For cell invasion assays, the filter membranes were coated with Matrigel (30 *μ*g, Sigma, USA). Cell migration assays did not require a coat of Matrigel in the upper chamber. Cells (1 × 10^5^) suspended in 200 *μ*L of serum-free medium were seeded onto the upper compartment of the Transwell chamber. The lower chamber was filled with medium containing chemoattractants (10% FBS for migration and invaded cancer cells) and various concentrations of PC-SPESII. After incubation for 24 h, the medium in the upper chamber was removed, and the filters were fixed with 70% ethanol for 10 min. The cells remaining on the upper surface of the filter membrane were then completely removed by wiping with a cotton swab, and the cells on the opposite surface of the filter membrane were stained with 0.5% Coomassie Brilliant Blue for 10 min. The migrated/invaded cells were then visualized and counted from six randomly selected fields (100x magnification) using an inverted microscope.

### 2.9. ELISA for Detection of Human MMP-2 and MMP-9 Protein Levels Secreted by Human Breast Cancer Cells

To measure human MMP-2 and MMP-9 secretion, MDA-MB-231 cells were treated with the indicated concentrations of PC-SPESII, and after 24 h the culture media was analyzed by ELISA using Human MMP-9 ELISA Kit from R&D Systems (Minneapolis, MN, USA) and Human MMP-2 ELISA Kit from RayBiotech. ELISA was done according to the instructions of the manufacturer. Each experiment was repeated three times.

### 2.10. Western Blot Analysis

Whole-cell lysate was loaded in each lane and separated by 10% or 8% SDS-PAGE. Protein expression was detected using primary antibody (1 : 1000~5000) and IRDye conjugated secondary antibody (1 : 10000~20000). Levels of MMP-2, MMP-9, TIMP-1, TIMP-2, uPA, uPAR, p38MAPK, p-p38MAPK, p-ERK1/2, p-SAPK/JNK, SAPK/JNL, and GAPDH were analyzed in this manner. Quantitative analysis of Western blotting was done using Alpha Ease FC (FluorChem FC2) software. Using the analysis tools, we calculated the density ratio of protein to GAPDH, the loading control.

### 2.11. Statistical Analyses

All data are expressed as means ± SD. Comparisons between groups were performed by Student's *t*-test and one-way analysis of variance (ANOVA). The level of significance was set at *P* < 0.05. 

## 3. Results

### 3.1. PC-SPESII Inhibits *In Vivo* Pulmonary Metastasis of MDA-MB-231 Cells in Nude Mice

**Figure 2:**
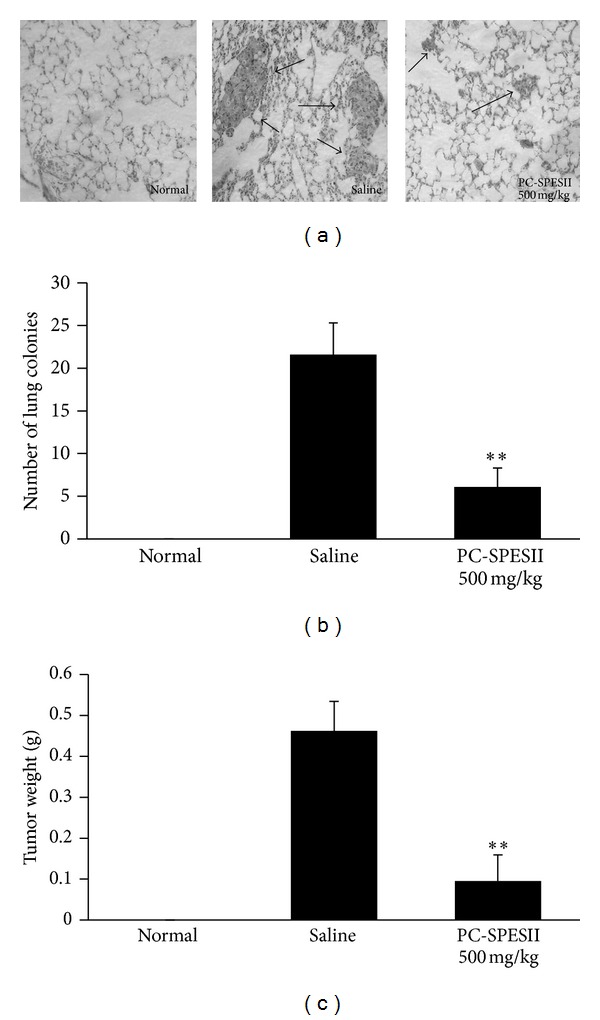
PC-SPESII inhibits pulmonary metastasis of MDA-MB-231 cells in Balb/c mice. Mice were divided into three experimental groups and were given drugs by oral gavage. The experiment was terminated 8 weeks after the initiation of therapy. Lungs were removed and fixed with Bouin's solution for 24 hours. Metastatic lesions on the lungs were counted under a dissecting microscope (100x magnification). (a) Histological appearances of representative lungs from normal, saline-treated, and PC-SPESII-treated mice are shown. (b) Quantification of metastatic lung nodules. (c) Tumor weights in grams of animals with different treatments were measured. **P < 0.01 compared to the saline group.

To determine whether PC-SPESII can inhibit human breast cancer metastasis, we examined the effects of PC-SPESII on spontaneous lung metastasis using MDA-MB-231 human breast cancer xenograftsin nude mice. Histological examination of the lung sections showed high levels of metastasized MDA-MB-231 cells in saline-fed mice (Figure 2(a)). The average number of tumor nodules was 21.60 ± 3.92 in the saline-treated group and 6.10 ± 2.33 in the PC-SPESII-treated group. These results indicated that PC-SPESII treatment significantly decreases tumor colonization in the lung compared with the saline group (*P* < 0.01; Figure 2(b)). Moreover, tumor weight was significantly inhibited in the PC-SPESII-treated group as shown in Figure 2(c) (*P* < 0.01). Together, these results strongly suggested that PC-SPESII can inhibit cancer metastasis and tumor growth. 

### 3.2. Side Effects of PC-SPESII in Nude Mice

**Figure 3:**
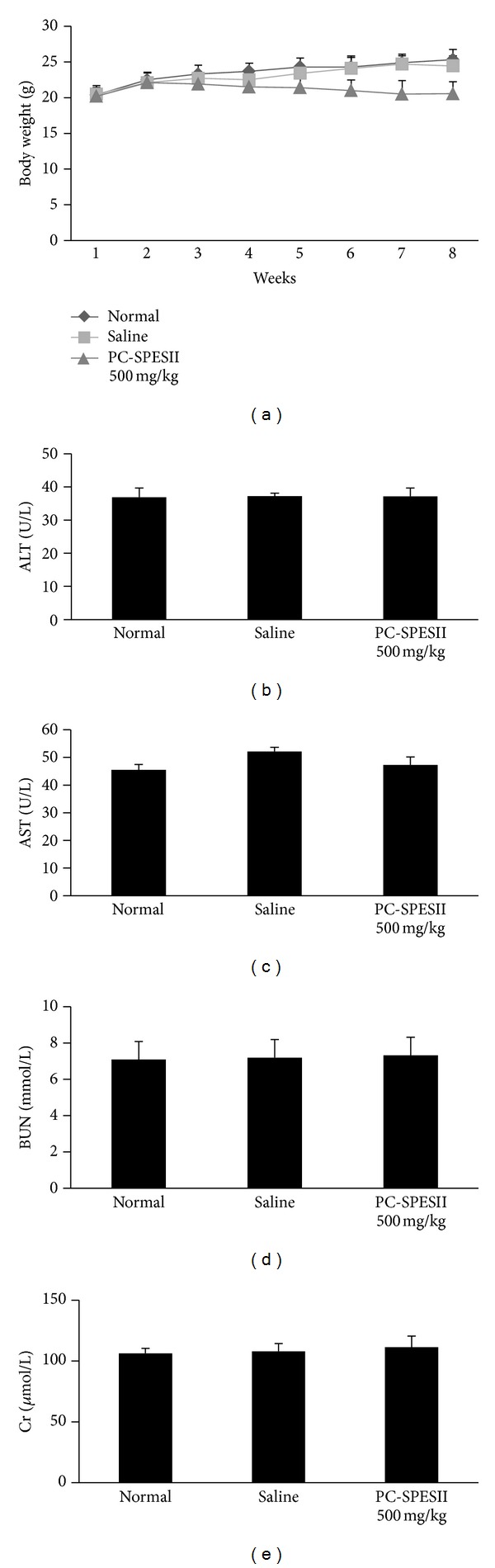
Effect of PC-SPESII on body weight, kidney function, and liver function in nude mice. Mice were treated with saline or PC-SPESII (500 mg/kg) for 8 weeks. (a) Body weights in grams of animals with different treatments were measured every week. (b) ALT, (c) AST, (d) BUN, (e) Cr and were measured using the colorimeter testing kit. According to the manufacturer's instructions, serum samples were measured at 510 nm, 510 nm, 510 nm, and 520 nm, respectively.

In order to detect the potential side effects of PC-SPESII, we measured the body weight of mice every week. As shown in Figure 3(a), there were no significant differences in body weight among the three experimental groups (*P* < 0.05). We further tested the effects of PC-SPESII on liver and kidney functions. We did not detect significant changes on ALT (Figure 3(b)), AST (Figure 3(c)), BUN (Figure 3(d)), and Cr (Figure 3(e)) among the PC-SPESII-treated group, normal group, and saline group (*P* < 0.05), indicating that liver and kidney functions were normal after PC-PSESII treatment. Together, these results suggest that PC-SPESII has no side effects in mice.

### 3.3. Effect of PC-SPESII on Human Breast Cancer Cell Viability

**Figure 4:**
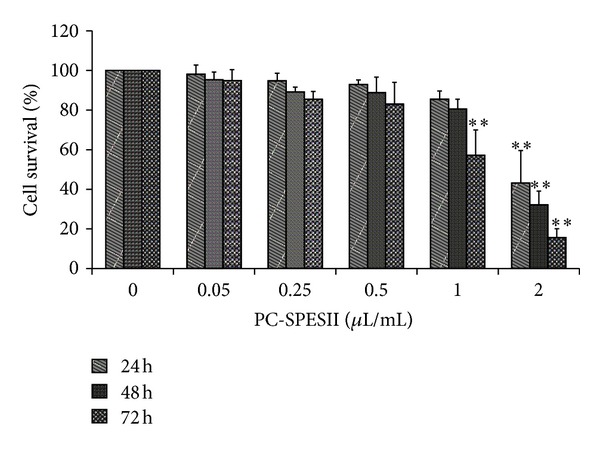
Effect of PC-SPESII on breast cancer cell viability. MTT assay was performed to measure cell survival (by percent) in response to PC-PSESII treatment. MDA-MB-231 cells were treated with the indicated amounts of PC-SPESII for 24, 48, or 72 hours. Results are presented as means ± SD of three independent experiments and SD are denoted by error bars (**P < 0.01 compared to untreated control).

In light of our findings *in vivo*, we further tested PC-SPESII *in vitro*. We first determined the effect of PC-SPESII on MDA-MB-231 cell viability by MTT assay. As shown in Figure 4, cell survival was inhibited after 72 h treatment with 1 *μ*L/mL PC-PSESII. Doubling the dose to 2 *μ*L/mL resulted in increased inhibition after 24 h, 48 h, and 72 h treatments. No significant differences were detected within 48 h at concentrations lower than 2 *μ*L/mL compared with untreated cells. Therefore, to test the effects of PC-SPESII on human breast cancer cell invasion and migration without confounding effects from cytotoxicity, nonlethal concentrations (<2 *μ*L/mL) and treatment times (<48 h) were used in subsequent experiments.

### 3.4. PC-SPESII Inhibits *In Vitro* Human Breast Cancer Cell Invasion

**Figure 5:**
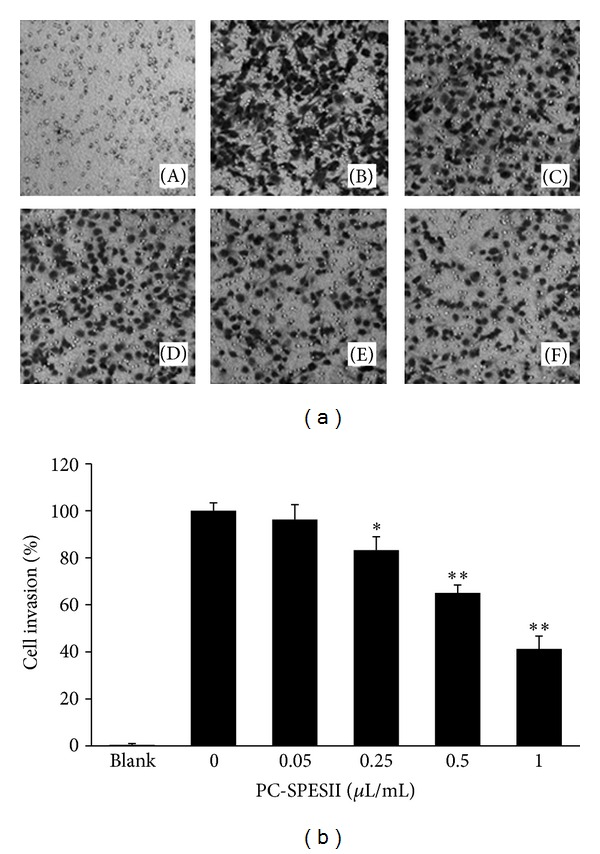
Effect of PC-SPESII on MDA-MB-231 cell invasion. (a) Transwell chamber was used for the invasion assay and images were taken at 200x magnification. The filter membranes were coated with Matrigel. MDA-MB-231 cells were treated with 0 (B), 0.05 (C), 0.25 (D), 0.5 (E), or 1 (F) *μ*L/mL of PC-SPESII for 24 hours. No cells were seeded in (A). (b) Stand and error bars represent three independent experiments and each experiment was performed in triplicate (*P < 0.05 and **P < 0.01 compared to untreated control).

Metastasis consists of sequential steps involving cancer cell invasion and migration. To study whether PC-SPESII has anti-invasion effects, MDA-MB-231 cell invasion properties were analyzed by Matrigel coated Transwell chambers in the presence of PC-SPESII. Indeed, the number of cell invasions through the Matrigel coated filter was dose dependently reduced by PC-SPESII (Figure 5(a)). Compared with the control group, the number of invaded cells from PC-SPESII-treated (0.25, 0.5, and 1 *μ*L/mL) samples was reduced by 17%, 35%, and 59% respectively (*P* < 0.01; Figure 5(b)). Thus, in addition to its inhibitory effect on cell viability at high concentrations, low concentrations of PC-SPESII inhibited the cell invasion potential of MDA-MB-231 cells *in vitro*. Notably, the inhibitory effects of PC-SPESII on cell invasion were not due to its cytotoxic effects because viability was barely affected at the concentration range tested (Figure 4).

### 3.5. PC-SPESII Inhibits *In Vitro* Human Breast Cancer Cell Migration

**Figure 6:**
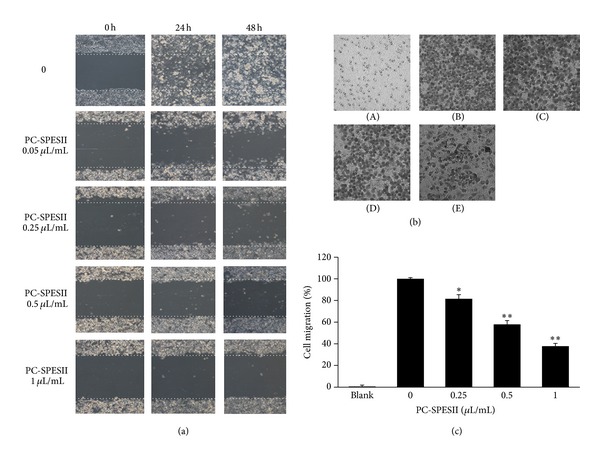
Effect of PC-SPESII on MDA-MB-231 cell migration. (a) Images of wound healing assays (100x magnification). Cells were seeded into 12-well cell culture plates, cultured in DMEM supplemented with 10% FBS, and allowed to grow to near confluence. Confluent monolayers were carefully wounded and the cellular debris was gently washed away with PBS. The wounded monolayer was reincubated in FBS-free DMEM containing 0, 0.25, 0.5, or 1 *μ*L/mL of PC-SPESII for 24 or 48 hours. (b) Transwell chamber was performed for the migration assay (200x magnification). MDA-MB-231 cells were treated with 0 (B), 0.25 (C), 0.5 (D), or 1 (E) *μ*L/mL of PC-SPESII for 24 hours during assay. No cells were seeded in (A). (c) Stand and error bars represent three independent experiments and each experiment was done in triplicate (*P < 0.05 and **P < 0.01 compared to untreated control).

We next tested whether PC-SPESII can inhibit the migration ability of MDA-MB-231 cells. We initially tested this by performing a wound-healing assay. Confluent cells were scraped with a sterilized tip and the remaining cells were allowed to migrate into the gap created in the absence or presence of PC-SPESII as shown in Figure 6(a). Remarkably, after 24 and 48 h incubation, the wound gap was wider in the PC-SPESII-treated (0.05, 0.25, 0.5, and 1 *μ*L/mL) groups than the untreated group, indicating that PC-SPESII inhibits MDA-MB-231 cell motility.

To corroborate these findings, we tested the effect of PC-SPESII on MDA-MB-231 cell motility by the Transwell chamber assay. As expected from the wound-healing assay, the number of cells migrating to the lower chamber was reduced in response to PC-SPESII treatment in a concentration-dependent manner (Figure 6(b)). Compared to the untreated group, the number of migrated cells from PC-SPESII-treated (0.25, 0.5, and 1 *μ*L/mL) groups was reduced by 19%, 42%, and 62%, respectively (*P* < 0.01; Figure 6(c)). Together, these data confirm that PC-SPESII inhibits MDA-MB-231 cell migration.

### 3.6. PC-SPESII Reduces MMP-2 and MMP-9 Secretion in MDA-MB-231 Cells

**Figure 7:**
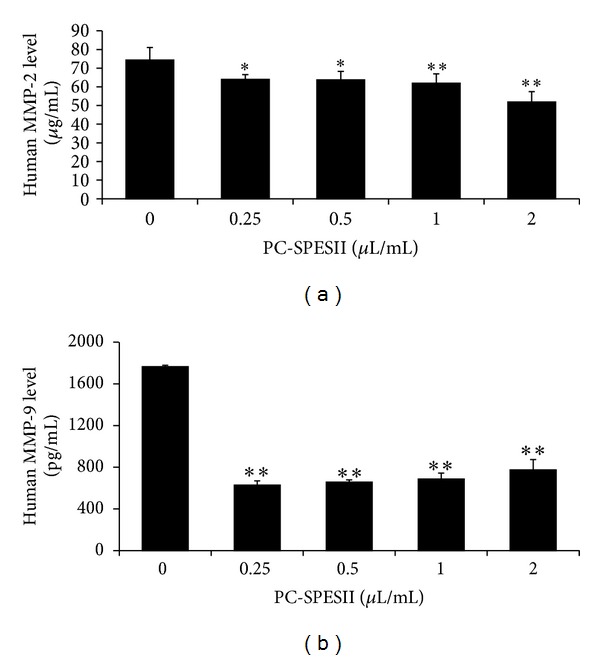
PC-SPESII reduces MMP-2 and MMP-9 extracellular secretion in MDA-MB-231 cells. MDA-MB-231 cells were treated for 24 h with the indicated concentrations of PC-SPESII. Then, each cell culture medium was collected and analyzed by ELISA using Human MMP-2 and MMP-9 ELISA Kit (a, b). ELISA was done according to the instructions from the manufacturer. Each experiment was repeated three times. *P < 0.05 and **P < 0.01 compared to untreated control.

Degradation of extracellular matrix and basement membrane are very important steps in cancer invasion and metastasis. MMP-2 and MMP-9 are the two important proteolytic enzymes involved in this process. Here, we tested secretion levels of human MMP-2 and MMP-9 from MDA-MB-231 cells with or without PC-SPESII treatment. As shown in Figures 7(a) and 7(b), PC-SPESII significantly inhibited MMP-2 (0.25 and 0.5 *μ*L/mL, *P* < 0.05; 1 *μ*L/mL, *P* < 0.01) and MMP-9 (*P* < 0.01) secretion into the medium in a dose-dependent manner. This result suggests that PC-SPESII-dependent inhibition of breast cancer metastasis may involve the degradation of extracellular matrix and basement membrane. 

### 3.7. PC-SPESII Regulates Proteolytic Enzymes

**Figure 8:**
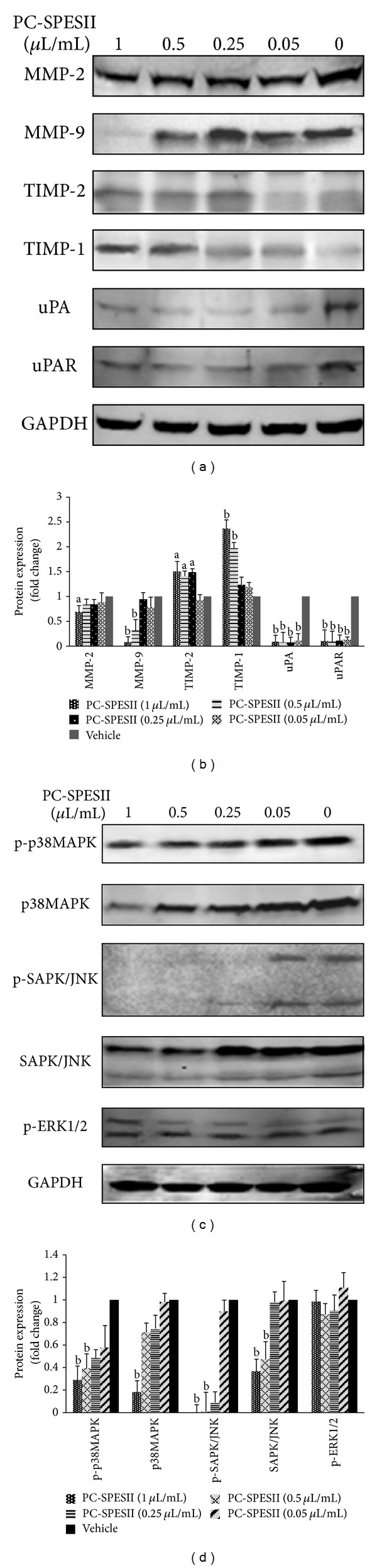
Changes in protein expression in response to PC-SPESII treatment. (a, c) MDA-MB-231 cells were treated with or without PC-SPESII for 24 h. Then Western blot analysis was performed using antibodies specific for MMP-2, MMP-9, uPA, uPAR, TIMP-1, TIMP-2, p38MAPK, p-p38MAPK, p-ERK1/2, p-SAPK/JNK, and SAPK/JNK. (b, d) The density ratio of proteins to GAPDH is shown as relative expression. Values are expressed in mean ± SD; three experiments were repeated with similar results. ^a^ *P* < 0.05 and ^b^ *P* < 0.01 compared to control.

To determine whether proteolytic protein expression is regulated by PC-SPESII, cells were treated with or without PC-SPESII for 24 h and whole-cell extracts were analyzed by Western blotting. As shown in Figures 8(a) and 8(b), MMP-2, MMP-9, uPA, and uPAR levels were decreased by varying degrees in response to 1 *μ*L/mL and other concentrations of PC-SPESII. Alternatively, TIMP-1 and TIMP-2 levels were significantly increased. Taken together, these data suggest that PC-SPESII-mediated inhibition of MDA-MB-231 cell migration, invasion, and metastasis is dependent on the degradation of extracellular matrix.

### 3.8. PC-SPESII Regulates Expression of Proteins Involved in the p38MAPK and SAPK/JNK Pathway

The MAPK and SAPK/JNK signaling pathway has been implicated in the regulation of various cellular processes including cancer cell metastasis. This pathway also regulates the expression of proteolytic proteins and ECM degradation. Thus, we tested the effect of PC-SPESII on MAPK levels and signaling by Western blotting. In response to PC-SPESII treatment, p38MAPK and SAPK/JNK expression were reduced while p-ERK1/2 expressions remained unchanged (Figures 8(c) and 8(d)). Phosphorylation of p38MAPK and SAPK/JNK was also reduced. The p38MAPK protein phosphorylated ratios were 0.59, 0.65, 0.55, and 1.60. The SAPK/JNK protein phosphorylated ratios were 0.90, 0.09, 0.02, and 0.01. These results confirmed that the signaling competency of these proteins was compromised. 

**Figure 9:**
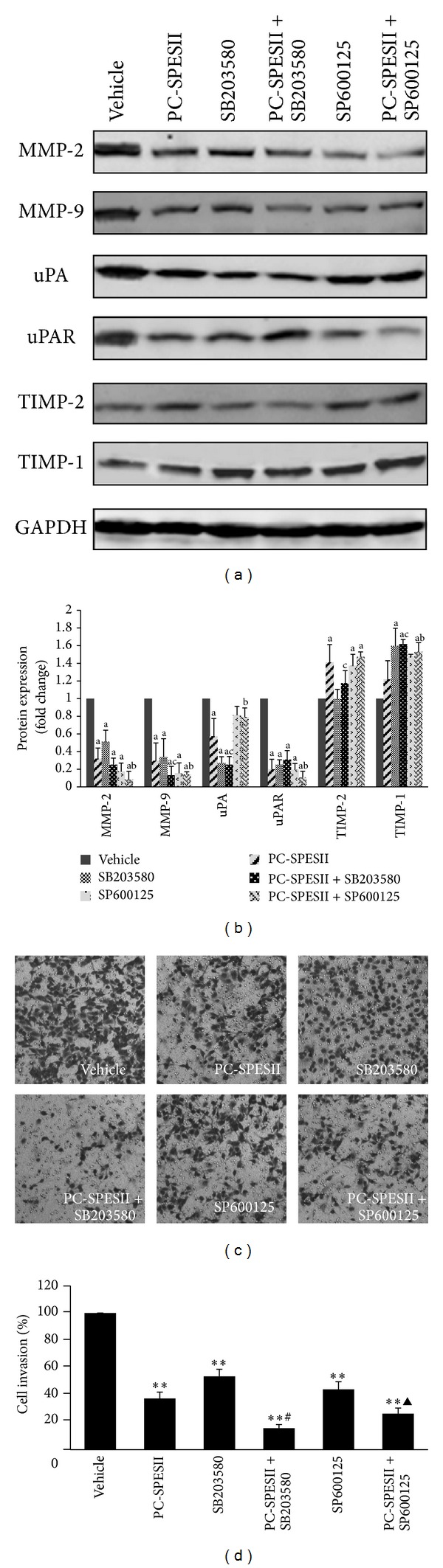
Protein expression and cell invasion after PC-SPESII treatment with or without p38MAPK and SAPK/JNK inhibitors. (a) MDA-MB-231 cells were treated with or without 1 *μ*L/mL PC-SPESII, 15 *μ*m/L SB203580, and 20 *μ*m/L SP600125 in the indicated combinations for 24 h. Then, Western blot analysis was performed and stained for the indicated epitopes. (b) The density ratio of proteins to GAPDH is shown as relative expression. Values are expressed as mean ± SD. Three experiments were repeated with similar results. ^a^ *P* < 0.01, ^b^ *P* < 0.05, and ^c^ *P* < 0.05 compared to control. (c, d) Transwell chamber was used for the invasion assay (100x magnification). The filter membranes were coated with Matrigel. Cells were treated according to the conditions in (a) for 24 h. Results are presented as mean ± SD of three independent experiments. **P < 0.01 compared to control; ^#^ *P* < 0.01 and ^▲^ *P* < 0.05 compared to 1 *μ*L/mL PC-SPESII treatment.

We hypothesized that pharmacological inhibition of p38MAPK and SAPK/JNK signaling would recapitulate the phenotypes associated with PC-SPESII treatment. To test this, we treated MDA-MB-231 cells with the p38MAPK-specific inhibitor (SB203580) and the SAPK/JNK-specific inhibitor (SP600125) alone or in combination with PC-SPESII. Interestingly, inhibiting p38MAPK or SAPK/JNK mimicked the effects we described in PC-SPESII-treated cells (Figure 9), indicating that PC-SPESII acts on these signaling pathways. Since the inhibitors and PC-SPESII act on the same pathway, we reasoned that cotreatment would amplify their effects. Compared to cells treated with PC-SPESII alone, expression of MMP-9, uPA, TIMP-1, and TIMP-2 was further reduced in cells cotreated with PC-SPESII and SB203580 or SP600125 (Figures 9(a) and 9(b)). Next, we tested the effect of cotreatment on cell invasion by the Transwell chamber assay. Again, PC-SPESII and the two inhibitors combined to further reduce cell invasion (Figures 9(c) and 9(d)). Taken together, these results suggest that the anti-invasion effect of PC-SPESII acts through the p38MAPK and SAPK/JNK pathway in MDA-MB-231 cells. 

## 4. Discussion

Most cancer patients do not die from local complications of their primary tumor growth, but rather from the development and spread of the tumor. Preventing and suppressing tumor invasion and metastasis is a promising means for decreasing the mortality of patients with malignant tumors. In recent years, studies performed on antitumor drugs are increasing. These include natural products that have been used as alternative treatments for treating certain cancers such as breast cancer [25, 26]. Despite the increased research in this field, there still remains a serious shortage of agents that target cancer cell metastasis [27].

This study focuses on the mechanism and antimetastatic effects of PC-PSESII, a Chinese herbal medicine. We demonstrated that PC-PSESII has high antimetastatic activity and low toxicity. Specifically, PC-PSESII suppressed pulmonary metastasis of human MDA-MB-231 breast cancer cells in nude mice. The average number of metastasized lung nodules and tumor weight in the PC-SPESII-treated group was significantly lower than in saline group (Figure 2). Despite its dramatic effects on cancer cell metastasis, PC-SPESII treatment did not alter body weight, kidney function, and liver function (Figure 3) *in vivo*. We also found concentration-dependent inhibition of MDA-MB-231 cell migration and invasion in response to PC-SPESII *in vitro* (Figures 5 and 6). These results indicated that PC-SPESII has potent antimetastatic activity *in vivo* and *in vitro* with no side effects.

 The ECM and BM are two barriers that hinder cancer cell invasion. The MMP family of zinc-dependent proteinases mediates ECM degradation. MMP-2 and MMP-9 are the key enzymes for type IV collagen degradation and are considered to be important for cancer invasion [28]. In cancer cells, MMP-2 and MMP-9 are controlled by their endogenous inhibitors TIMP-1 and TIMP-2 [29]. Therefore, decreasing MMP activity while increasing TIMP activity could inhibit cancer cell invasion and metastasis [30]. In order to investigate the mechanisms of the antimigration and anti-invasion effects of PC-SPESII, we examined the regulation of MMP and TIMP activity in MDA-MB-231 cells. Our data showed that PC-SPESII significantly inhibited MMP-2 and MMP-9 secretion (Figure 7) and expression and increased TIMP-1 and TIMP-2 levels (Figure 8(a)), consistent with its inhibitory effects on metastasis. 

uPA is another important ECM proteinase. It is a serine protease that converts plasminogen to plasmin, which directly mediates cancer cell invasion by degrading matrix proteins such as collagen IV, fibronectin, and laminin or indirectly by activating MMP-2, MMP-3, MMP-9, and uPA [31]. It is well documented that overexpression of uPA in breast cancers is a strong indicator of poor prognosis. uPAR focuses uPA activity on the cell membrane, thus regulating cell surface-associated plasminogen proteolysis by uPA [32]. Thus, we studied the effects of PC-SPESII on uPA and uPAR in MAD-MB-231 cells. The results showed that PC-SPESII decreased uPA and uPAR expression (Figure 7(a)). Taken together, our data indicates that PC-PSESII exerts its effects through regulating the balance between MMP and TIMP expression and decreasing uPA and uPAR expression* in vitro.*

The MAPK pathway is upstream of MMP activity and ECM degradation. It has been reported that the overexpression and phosphorylation of MEK and ERK may play an important role in the development of human breast cancer [33]. Tetraspanin CD9 activates p38MAPK, which induces MMP expression and activates JNK and c-Jun pathways in human melanoma cells [34]. Previous studies have shown that p38MAPK phosphorylation occurs in 20% of primary breast carcinomas and may be associated with poor outcomes in patients with lymph node-positive breast carcinoma [35]. P38MAPK could promote breast cancer progression by upregulating uPA expression, suggesting that phosphorylated p38MAPK and uPA expression could serve as biomarkers for breast cancer prognoses [20]. A natural product, Butein, inhibits the migration and invasion of SK-HEP-1 human hepatocarcinoma cells by suppressing the ERK, JNK, p38, and uPA signaling pathways [36]. Constitutive p38alpha MAPK activity is required for increased uPAR expression and matrix invasion by breast cancer cells [37]. Downstream from MAPK signaling, JNK activation helps regulate cancer cell invasion and expression of MMP-1, MMP-2, and MMP-9. Accordingly, inhibiting JNK decreases cancer cell invasion [38]. 

In this study, we confirmed that PC-SPESII functions by inhibiting the p38MAPK and SAPK/JNK pathway without altering ERK1/2 phosphorylation. In order to further assess the role of p38MAPK and SAPK/JNK in PC-SPESII treatment, MDA-MB-231 cells were treated with the p38MAPK-specific inhibitor, SB203580, and the SAPK/JNK-specific inhibitor, SP600125, alone or in combination with PC-SPESII. The results showed that the effects of PC-SPESII combined with inhibitors were significantly strengthened (Figures 9(a) and 9(b)), suggesting that PC-SPESII regulates the proteolytic enzyme via the p38MAPK and SAPK/JNK pathway in MDA-MB-231 cells. 

## 5. Conclusion

PC-SPESII inhibits human breast cancer MDA-MB-231 cell migration, invasion, and metastasis through *in vitro* and* in vivo* studies. PC-PSESII regulates secretion and expression of proteolytic enzymes by targeting the p38MAPK and SPK/JNK pathway. The potent antimetastatic effect and low toxicity of PC-PSESII suggest that this Chinese herbal remedy has a high therapeutic potential for metastatic breast cancer. 

## Figures and Tables

**Table 1:** Quality control standardization for PC-SPESII.

Compounds (marker)	Refers to	Quality criterion (lowest amounts tolerated in 19 g of PC-SPESII crude extract)
Baicalin	Huangqin	15 mg

Indirubin	Daqingye	12 mg

Oridonin	Donglingcao	10 mg

Notoginsenoside	Sanqi	8 mg

Ganoderma lucidum polysaccharides	Lingzhi	1 mg

Chrysanthemum yellow ketone	Juhua	4 mg

Glycyrrhizic acid	Gancao	8 mg
